# Transcriptional Characteristics of *IDH*-Wild Type Glioma Subgroups Highlight the Biological Processes Underlying Heterogeneity of *IDH*-Wild Type WHO Grade IV Gliomas

**DOI:** 10.3389/fcell.2020.580464

**Published:** 2020-10-22

**Authors:** Yu-Zhou Chang, Guan-Zhang Li, Bo Pang, Ke-Nan Zhang, Xiao-Hui Zhang, Yong-Zhi Wang, Zhong-li Jiang, Rui-Chao Chai

**Affiliations:** ^1^Department of Molecular Neuropathology, Beijing Neurosurgical Institute, Beijing Tiantan Hospital, Capital Medical University, Beijing, China; ^2^Department of Neurosurgery, Beijing Neurosurgical Institute, Beijing Tiantan Hospital, Capital Medical University, Beijing, China; ^3^China National Clinical Research Center for Neurological Diseases, Beijing Tiantan Hospital, Capital Medical University, Beijing, China; ^4^Chinese Glioma Genome Atlas Network, Beijing, China

**Keywords:** *isocitric dehydrogenase*-wild type glioma, molecular oncology research, cell cycle, extracellular matrix, immune microenvironment

## Abstract

*Isocitric dehydrogenase (IDH)*-wild type diffuse gliomas, which have a poorer prognosis than their *IDH*-mutant counterparts, are also accompanied with high heterogeneity. Here, we aimed to identify the key biological processes associated with the three groups of *IDH*-wild type diffuse gliomas in 323 patients. By The Consortium to Inform Molecular and Practical Approaches to CNS Tumor Taxonomy (cIMPACT-NOW) update 3 recommendation, the three groups are Group A, diffuse astrocytic glioma, World Health Organization (WHO) grade II/III; Group B, diffuse astrocytic glioma, with one (or more) of the three genetic alterations: *TERT* promoter mutation, *EGFR* gene amplification, gain of chromosome 7 combined with loss of chromosome 10, WHO grade IV; and Group C, glioblastoma, WHO grade IV. Consistent with their histologic and genetic molecular features, we successfully identified that biological activities associated with “cell cycle” and “cell mitosis” are significantly elevated in Group B compared with Group A; microenvironment-related hallmarks “angiogenesis” and “hypoxia,” and biological processes of “extracellular matrix,” “immune response,” and “positive regulation of transcriptional activities” were more enriched in Group C than Group B. We also constructed a nine-gene signature from differentially expressed genes among the three groups to further stratify the WHO grade IV gliomas (Groups B and C) whose survival cannot be clearly stratified by current classification systems. This signature was an independent prognosis factor for WHO grade IV gliomas and had better prognostic value than other known factors in both training and validation dataset. In addition, the signature risk score was positively correlated with the amount of infiltrated immune cells, expression of immune checkpoints, and the genes enriched in biological processes of “immune response,” “cell cycle,” and “extracellular matrix.” The bioinformatic analysis results were also validated by immunohistochemistry and patient-derived cell proliferation assay. Overall, our findings revealed the key biological processes underlying the new classifications of *IDH*-wild type diffuse glioma. Meanwhile, we constructed a signature, which could properly stratify the prognosis, cell proliferation activates, extracellular matrix-mediated biological activities, and immune-microenvironment of *IDH*-wild type WHO grade IV gliomas.

## Introduction

Diffuse glioma is one of most common types of malignant primary tumor in the CNS (Jiang et al., 2016). According to the WHO classification of tumors of the CNS, diffuse gliomas are classified into grades II to IV based on histologic features (Jiang et al., 2016; Louis et al., 2016). Currently, the prognosis of gliomas is still dismal, and the overall survival (OS) for the glioblastoma (GBM, WHO grade IV) is only about 14–16 months even after receiving comprehensive therapy (Chai et al., 2018, 2019b). Recent advances in the molecular pathology of gliomas have brought a glimmer of hope for improving the prognosis of glioma through precision therapy (Hegi et al., 2004; Yan et al., 2009; Louis et al., 2016; Bell et al., 2018; Gao et al., 2018; Hu et al., 2018). In addition to histologic features, molecular features, such as *IDH* and 1p/19q codeletion statues, had also been included in the 2016 WHO classification (Louis et al., 2016). *IDH*-wild type gliomas, including diffuse astrocytic gliomas (WHO grade II/III) and GBM (WHO grade IV), have a poorer prognosis compared with the same histologic *IDH*-mutant gliomas (Chai et al., 2020). Recently, growing evidence indicated that some *IDH*-wild type astrocytic gliomas had similar molecular features and prognosis of *IDH*-wild type GBM (Yang et al., 2016; Aibaidula et al., 2017; Pekmezci et al., 2017; Wijnenga et al., 2017), suggesting that the specific subgroup of *IDH*-wild type glioma still needs to be further classified.

Recently, the update three of cIMPACT-NOW recommend that a partial of *IDH*-wild type diffuse astrocytic tumors should also be determined as WHO grade IV, if there is one (or more) of the three genetic alterations: *TERT* promoter mutation, *EGFR* gene amplification, gain of chromosome 7 combined with the loss of chromosome 10 (combined 7 + /10−) (Brat et al., 2018). This updated criterion promoted our understanding in *IDH*-wild type gliomas based on their genetic feature, highlighting the value of molecular features in stratifying tumors with similar histologic features. However, the biological processes underlying this updated criterion are still unclear.

In addition to genetic features, the transcription characteristic of a subgroup of tumors is also indispensable for understanding the biological characteristics of these tumors (Verhaak et al., 2010; Sun et al., 2014; Ceccarelli et al., 2016; Chai et al., 2019c,d). Our previous reports also indicated that transcriptional alterations between tumors with different molecular features is valuable for further understanding biological processes that contribute to the progression of gliomas (Chai et al., 2019c,d). Thus, these update criteria also provide a clue for understanding the key biological processes, which may contribute to the progression of *IDH*-wild type gliomas. Compared with genetic alterations, which take into account only the tumor itself, transcriptional features could also reflect alterations in a tumor microenvironment, such as immune infiltrating information (Ceccarelli et al., 2016; Griveau et al., 2018; Vidyarthi et al., 2019; Wu et al., 2020). In addition, growing evidence indicates that transcriptional features could serve as robust stratifying tools to further stratify glioma (Pomeroy et al., 2002; Ceccarelli et al., 2016; Chai et al., 2018, 2019b,c,d; Wu et al., 2020). Thus, characterizing transcriptome characteristics of cIMPACT-NOW update three subgroups of *IDH*-wild type gliomas has important biological and clinical significance.

In this study, we aimed to characterize the transcriptome difference among different subgroups of *IDH*-wild type gliomas after the cIMPACT-NOW update three in two independent datasets. We also revealed the key biological processes and tumor-environment alterations underlying this updated criterion through annotating the biological functions of these DEGs. Finally, we also successfully further stratified the *IDH*-wild type WHO Grade IV gliomas through a transcriptional signature derived from these DEGs.

## Materials and Methods

### Sample Collection

Given that *EGFR* amplification and chromosome 7 + /10− was not tested for samples in our CGGA dataset, we enrolled 224 patients of TCGA database as finding dataset, according to the following criteria: (a) adult patients with the entire clinic, pathological, and transcriptome information (b) pathologic diagnosis as *IDH*-wild type glioma. All these clinic manifestations and pathologic diagnosis information were downloaded from TCGA official website^1^ (Ceccarelli et al., 2016). We separated these gliomas into three subgroups: Group A, diffuse astrocytic glioma without any of *EGFR* amplification, or combined chromosome 7 + /10− and *TERT* promoter mutant; Group B, diffuse astrocytic glioma with either of *EGFR* amplification, combined chromosome 7 + /10− or *TERT* promoter mutant; and Group C, glioblastoma.

To verify the finding in TCGA dataset, we also enrolled 99 *IDH*-wild type patients in our CGGA database (Hu et al., 2018; Chai et al., 2019c), including 27 diffuse astrocytic gliomas whose *TERT* promoter status had been tested by pyrosequencing as previously reported (Chai et al., 2020), and we classified these patients into three groups: Group A, diffuse astrocytic glioma without *TERT* promoter mutant; Group B, diffuse astrocytic gliomas with *TERT* promoter mutant; and Group C, glioblastoma.

Clinicopathological information for all cases used in this study is summarized in Supplementary Table 1. This study was approved by the institutional review board of Beijing Tiantan Hospital.

### Selection and Functional Analysis of Differentially Expressed Genes

*t*-Test was used to evaluate the DEGs when comparing Group A with Group B and Group B with Group C (*P*-value < 0.05, fold change > 2 or fold change < 0.5). GSEA was performed to investigate the tumor-related hallmark enrichment in the comparison between (a) Group A and Group B, (b) Group B and Group C as previously reported (Chai et al., 2019c). Metascape online website^2^ was used to perform GO and KEGG pathway enrichment analyses of both the two DEGs in TCGA and CGGA database to identify the biological characteristics of different groups (Zhou et al., 2019). The genes included in the intersection of both two DEGs were further retained if they were highly related to the survival of patients (*P*-value < 0.05). Finally, 16 genes were selected for further analysis.

### Construction of Signature and Bioinformatics Analysis

The LASSO Cox regression algorithm was applied to these 16 differentially expressed genes in all TCGA *IDH*-wild type WHO grade IV gliomas. A total of nine genes were selected to build the risk signature, and the coefficients and normalized expression levels of these genes were used together to calculate risk scores in both the training database (TCGA database) and the validation database (CGGA database) according to the following formula: Risk score = (expr_gene__1_ × coefficient_gene__1_) + (expr_gene__2_ × coefficient_gene__2_) + … + (expr_genen_ × coefficient_genen_) (Chai et al., 2019a,b). Patients in the TCGA and CGGA databases were divided into high- and low-risk groups by their median risk score, respectively.

The Pearson correlation was used to identify genes that were significantly correlated with the risk score. GSEA and GO analysis were used to analyze the functional annotation of the correlated genes. The ESTIMATE algorithm was performed to calculate the fraction of stromal and immune cells with R package “estimate.” Cibersort was used to estimate the proportion of immune cell types in a mixed cell population online^3^ (Newman et al., 2015).

### Immunohistochemistry

We performed IHC in *IDH*-wild type glioma tissues obtained from the CGGA database. RNA-seq data of these patients were obtained to calculate their risk score. We performed IHC staining of cell proliferation and transcription-related proteins, including Ki-67 (1:100, ZSGB-Bio, Beijing, China), *CDK4* (cyclin-dependent kinase 4), *MYC* (*MYC* proto-oncogene), *CDK6*, *CDC20* (cell division cycle 20), *CD31* (platelet and endothelial cell adhesion molecule 1), and *CD14*. In brief, sections were deparaffinized and boiled with citrate antigen retrieval buffer. Then the primary antibodies (anti-*CDK4*, 1:150 dilutions, Abcam, Cambridge, United Kingdom; anti-*MYC*, 1:150, Abcam; anti-*CDK6*, 1:150 dilutions, Abcam; anti-*CDC20*, 1:150, Abcam; anti-*CD31*,1:100 Abcam; anti-*CD14*, 1:150, Abcam) were used to incubate the section overnight at 4°C. The sections were then incubated with the appropriate secondary antibodies (1:100, ZSGB-Bio, Beijing, China) at room temperature for 1 h.

### Patient-Derived Cells and CCK-8 Assay

Patient-derived cells (PDCs) were obtained from fresh surgical specimens of human primary GBMs and cultured as tumorspheres in DMEM/F12 medium supplemented with B27 supplement (Life Technologies), and bFGF and EGF (20 ng/ml each). Cell proliferation was studied by the CCK-8 kit (Dojindo Laboratories, Kumamoto, Japan) according to the manufacturer’s protocol as previously reported (Aibaidula et al., 2017). PDCs (3,000) were plated in each well of 96-well plates and cultured in the medium for 4 days. At the predesigned time point, 10 μl of CCK-8 reagent was added to the medium of six-repeated wells. The absorbances at 450 and 630 nm of the medium were measured after incubation for 2 h.

### Statistical Analysis

Student’s *t*-test and one-way ANOVA analyses were performed to compare the gene expression levels, risk scores, and other factors among different subgroups. The Kaplan–Meier method was used to compare the OS of patients in the low- and high-risk group. R package “pROC” was used to evaluate the prediction efficiency for 14.5-month survival and 3-year survival. The distribution difference of age was tested by the non-parametric test between the two risk groups, and χ^2^ tests were used to compare the distribution of other clinicopathological features in TCGA and CGGA databases. Two-tailed Student’s *t*-test was performed to compare the risk scores in patients grouped by other clinical or molecular pathological characteristics. In Pearson correlation analysis, Pearson coefficient > 0.5 and Bonferroni corrected *P* < 0.01 is defined as positively related and Pearson coefficient < −0.5, and Bonferroni corrected *P* < 0.01 is defined as negatively related. All statistical analyses were conducted using R v3.6.1^4^, SPSS 26.0 (SPSS, Inc., Chicago, IL, United States) and Prism 8 (GraphPad Software, Inc., La Jolla, CA, United States). *P* < 0.05 was considered significant in all analyses.

## Results

### Different Transcriptional Profile Among Three Subgroups of *Isocitric Dehydrogenase*-Wild Type Gliomas

**FIGURE 1:**
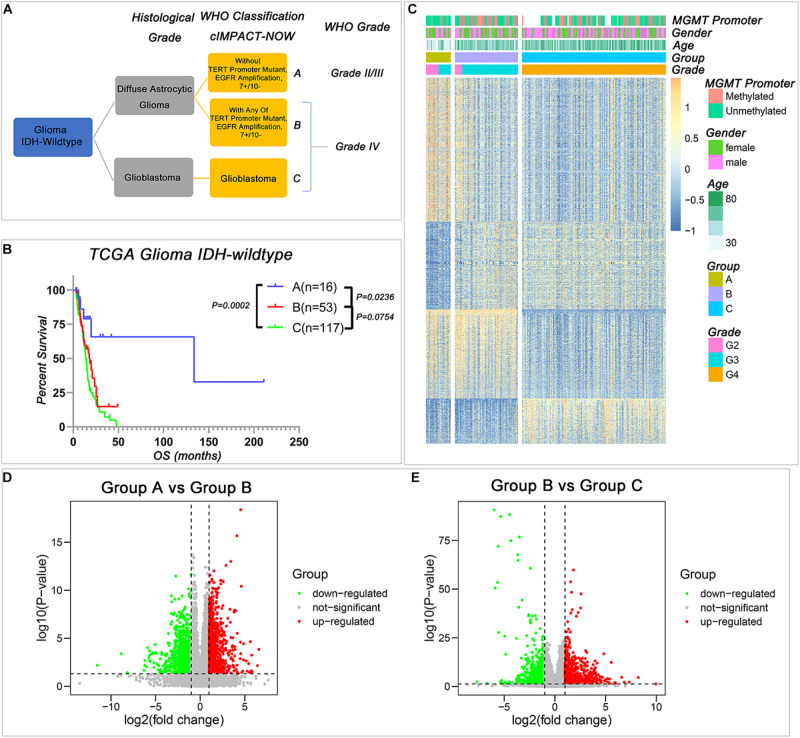
The transcriptional profile is different among the three *IDH*-wild type glioma groups of The Consortium to Inform Molecular and Practical Approaches to CNS Tumor Taxonomy (cIMPACT-NOW update 3). **(A)** The classification of *IDH*-wild type glioma according to cIMPACT-NOW update three recommendation **(B)** Kaplan–Meier overall survival (OS) curves for *IDH*-wild type glioma patients stratified by three groups. **(C)** Heatmap showing the expression pattern of DEGs among three groups of *IDH*-wild type gliomas. **(D,E)** The volcano plot of DEGs between Group A and Group B **(D)**, Group B and Group C **(E)**. *P*-value was calculated by two-tailed unpaired *t*-test.

As shown in Figure 1A, based on the cIMPACT update 3, *IDH*-wild type gliomas could be divided into three groups: Group A diffuse astrocytic gliomas without *TERT* promoter mutation, *EGFR* amplification, and combined 7 + /10−, WHO grade II/III; Group B, diffuse astrocytic gliomas with any of *TERT* promoter mutation, *EGFR* amplification, or combined 7 + /10−, WHO grade IV (simplified as *IDH*-wild type WHO grade IV gliomas with molecular feature of GBM); and Group C, GBM, WHO grade IV. To understand the differences among these three subgroups, we compared the survival of Group A, Group B, and Group C in TCGA dataset, and the results indicated that Group A survival was significantly longer than the other two groups, but the survival of WHO grade IV tumors cannot be stratified by Group B and Group C (Figure 1B). Then, we compared the gene expression levels among these three groups, and we successfully identified a large number of genes that differentially expressed among these three groups (Figures 1C–E).

Compared with Group A, there were 842 genes upregulated and 1,378 genes downregulated in Group B (Figure 1D and Supplementary Table 2). Compared with Group B, there were 755 genes upregulated, and 1,208 genes downregulated in Group C (Figure 1E and Supplementary Table 3). The similar results could also be observed in the CGGA database when we used the *TERT* promoter status to stratify the *IDH*-wild type diffuse astrocytic gliomas (Supplementary Tables 4, 5). These differentially expressed genes provided an effective way to understand the biological characteristics of these different groups of gliomas.

### The Biological Functions of Differentially Expressed Genes Among Three Subgroups

**FIGURE 2:**
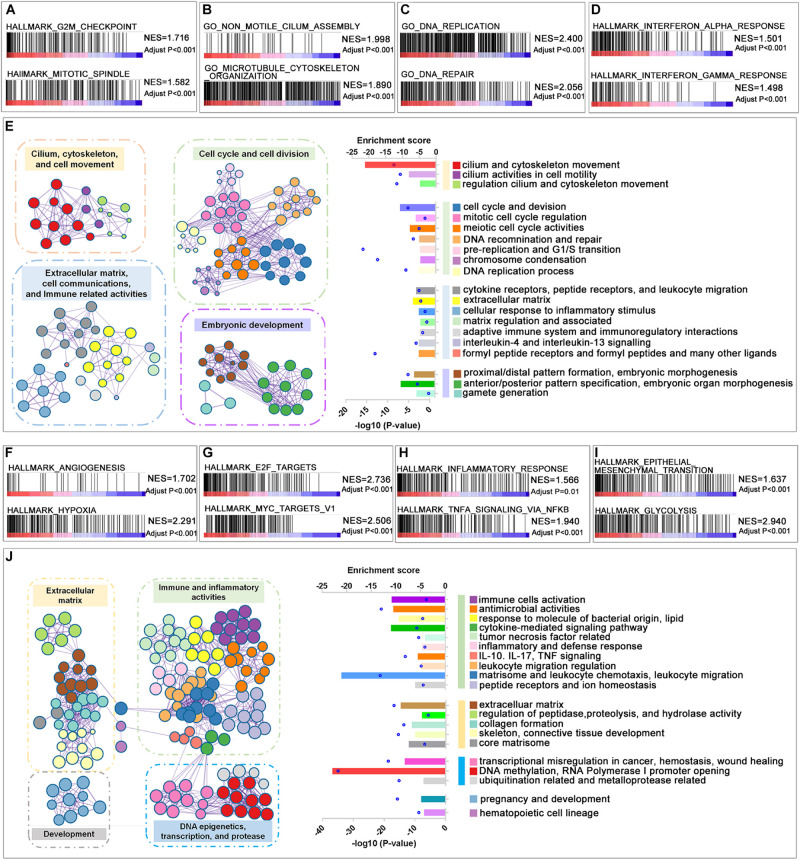
Function annotation of upregulated genes in Groups B and C. **(A–D)** Gene set enrichment analysis (GSEA) revealed the malignant tumor hallmarks and biological processes were enriched in Group B compared with Group A. **(E)** The GO analysis of upregulated DEGs in Group B compared with Group A. **(F–I)** GSEA revealed the hallmarks enriched in Group C compared with Group B. **(J)** The GO analysis of upregulated differentially expressed genes (DEGs) in Group C compared with Group B. Adjusted *P*-value was calculated by the permutation test in the GSEA analysis. The *P*-value in the GO analysis was calculated by Metascape online tools.

To dissect the biological differences among the three groups, we annotated the functions of the DEGs between Group B and Group A, and Group C and Group B, respectively. In the comparison between Group A and Group B, the GSEA results revealed that the cell cycle-related items “G2M checkpoint” and “mitotic spindle” (Figure 2A), extracellular matrix-related items “motile cilium assembly” and “microtubule cytoskeleton organization” (Figure 2B), DNA-related items “DNA replication” and “DNA repair” (Figure 2C) and immune response-related items “interferon alpha response” and “interferon gamma response” (Figure 2D) were enriched in Group B. Similar results could be found in a functional network of “Metascape analysis” (Zhou et al., 2019). Compared with gliomas in Group A, we noticed that the upregulated genes were mainly enriched in the biological processes or activities of “cilium, cytoskeleton, and cell movement,” “cell cycle and cell division,” “extracellular matrix, cell communication, and immune response,” and “embryonic development” (Figure 2E and Supplementary Table 6). These enriched functions in tumors of Group B reflected the impact of genetic variation, which included *TERT* promoter mutation, *EGFR* amplification, and combined 7 + /10−, on the tumor with similar histologic features. These results suggested that LGG with molecular features of GBM has more intensive activities of cell invasion and proliferation, and this also may be one of the reasons that patients with tumors in Group B had worse survival than Group A.

In the comparison between Group B and Group C, which have similar genetic alterations but distinct histologic features, the microenvironment-related hallmarks “angiogenesis” and “hypoxia” (Figure 2F), the transcriptional activity-related hallmarks “*E2F* targets” and “*MYC* targets v1” (Figure 2G), immune response-related hallmarks “inflammatory response” and “TNFA signaling via NFKB” (Figure 2H), and malignant progression-related hallmarks “epithelial mesenchymal transition,” and “glycolysis” (Figure 2I) were highly enriched in Group C. These observations are consistent with their differences in histologic features to some extent. The increased activities of “angiogenesis” may contribute to the microvascular proliferation and/or necrosis features of tumors of Group C. In turn, microvascular proliferation and/or necrosis may also result in elevated immune-related activities in tumors.

Consistently, we also observed that the elevated genes in Group C were enriched in “extracellular matrix,” “immune and inflammatory activities,” and “DNA epigenetics, transcription, and protease” in the functional networks (Figure 2J and Supplementary Table 7). We also analyzed the functions of genes that differentially expressed between *TERT* promoter mutant, *IDH*-wild type diffuse astrocytic gliomas (Group B), and GBM (Group C) in CGGA dataset (Supplementary Figures 1A–F). A similar result could be observed that genes upregulated in GBM mainly enriched in immune-related functions and extracellular matrix-related functions.

Interestingly, we noticed that downregulated genes in both comparisons, Group B with Group A and Group C with Group B, were enriched in terms of “synaptic function” and “transmembrane signal” in both TCGA and CGGA database (Supplementary Figures 2A–F). This suggested that normal synaptic components or activities were decreased along with the increasing malignant of *IDH*-wild type gliomas.

### Identification of Nine-Gene Transcriptional Signature From the Differentially Expressed Genes to Further Stratify *IDH*-Wild Type WHO Grade IV Gliomas

**FIGURE 3:**
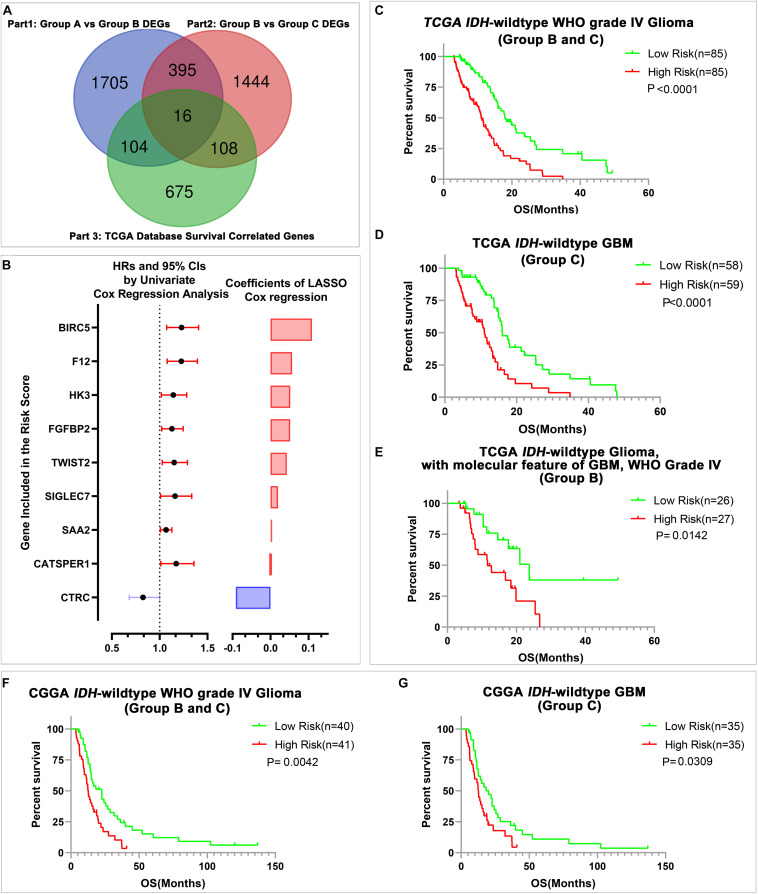
The construction of risk signature and its prediction value in *IDH*-wild type WHO grade IV gliomas. **(A)** The Venn diagram of candidate genes for LASSO analysis. **(B)** The nine genes included in the signature. Their HRs and 95% CIs were calculated by univariate Cox regression analysis, and the coefficients were calculated by multivariate Cox regression analysis using LASSO. **(C–E)** Kaplan–Meier OS curves of patients stratified by the median risk scores in all *IDH*-wild type WHO grade IV glioma (Group B and Group C), *IDH*-wild type glioblastoma (GBM) (Group C), and *IDH*-wild type glioma, WHO IV with the molecular feature of GBM (Group B) in TCGA database. **(F,G)** Kaplan–Meier OS curves of patients stratified by the median risk scores in all *IDH*-wild type WHO grade IV gliomas (Group B and Group C), and *IDH*-wild type GBM (Group C) in the Chinese Glioma Genome Atlas Network (CGGA) database.

Considering that transcriptional activities could not only reflect the genetic alterations of gliomas but also could represent the microenvironment differences among different gliomas. We sought to develop a stratification model with a small number of representative genes to further stratify the *IDH*-wild type WHO Grade IV gliomas (Group B and Group C) whose prognosis cannot be clearly stratified by current classification systems. Among the genes that changed consistently in both comparisons, Group B with Group A and Group C with Group B, we figured out that 16 genes significantly correlated with the survival of all *IDH*-wild type WHO grade IV gliomas in TCGA dataset (Figure 3A). Then, we applied the LASSO Cox regression algorithm to these 16 genes (Supplementary Figure 3A). Consequently, nine genes were selected to build the risk signature, and the coefficients of these genes were used to calculate the risk scores (Figure 3B and Supplementary Table 8).

Then, we evaluated the stratification ability of the signature in all *IDH*-wild type WHO grade IV gliomas (Group B and Group C). We divided patients into high- and low-risk groups in various groups of gliomas by their respective median risk score. We found that patients with high-risk scores had significantly shorter OS than patients with low-risk scores in all *IDH*-wild type WHO grade IV gliomas (Groups B and C) (Figure 3C), and its subgroup *IDH*-wild type GBM (Group C) (Figure 3D) and diffuse astrocytic gliomas, *IDH*-wild type, with molecular features of glioblastoma, WHO grade IV (Group B, Figure 3E) in TCGA dataset. The similar results could also be validated in the CGGA dataset, and risk scores could stratify the survival of all *IDH*-wild type WHO grade IV gliomas (Groups B and C) (Figure 3F) and *IDH*-wild type GBM (Group C) (Figure 3G). There is also a trend that patients with high-risk scores had shorter OS than those with low-risk scores in 10 patients with *IDH*-wild type and *TERT* promoter mutant diffuse astrocytic gliomas (Group B) in CGGA dataset, and insignificant *P*-value may be caused by the limited number of cases (Supplementary Figure 3B).

### The Signature Served as an Independent Prognostic Factor With Better Prognostic Prediction Efficiency Than Known Factors

**FIGURE 4:**
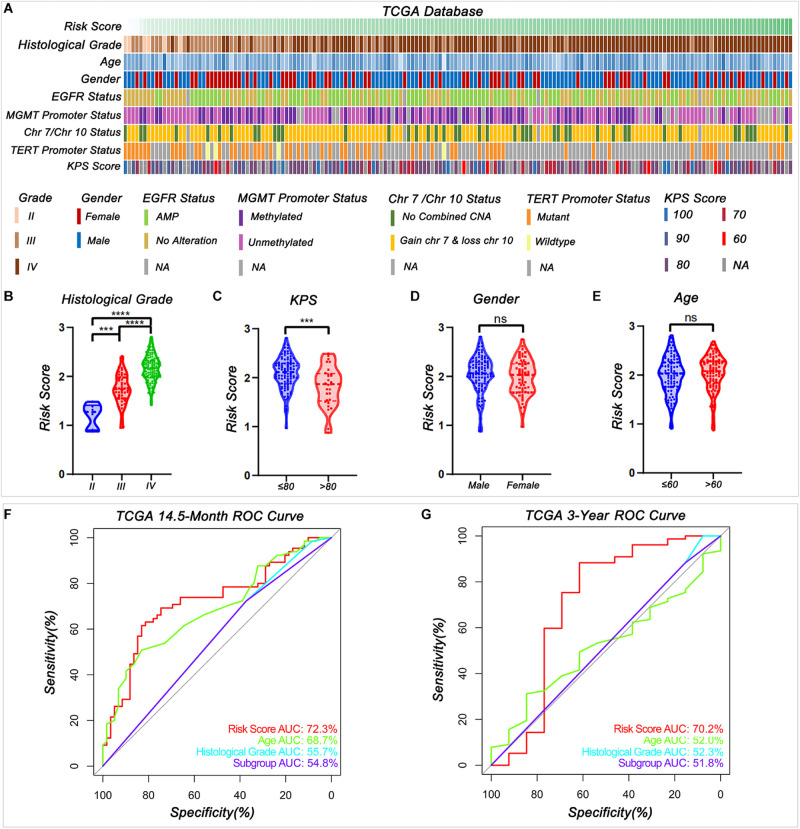
Relationship between the signature risk score and the clinic characteristics. **(A)** The distribution of clinic characteristics arranged by the increasing risk score. **(B–E)** The distribution of risk scores was stratified by the clinic characteristics (***P < 0.001, ****P < 0.0001, n.s., not significant). (**F–G**) Receiver operating characteristic (ROC) curves showed the predictive efficiency of risk score, age, histological grade according to Louis et al. (2016) summary, and subgroup according to cIMPACT-NOW update three recommendation on 14.5-month and 3-year survival in TCGA database.

To study the relationship between the risk score and clinicopathological characteristics, we arranged patients according to their risk score, and the landscape of other clinicopathological features was shown in the heatmap (Figure 4A and Supplementary Tables 9, 10). The results indicated that higher histological grade and lower KPS are mainly distributed in cases with higher risk scores. We also observed that the risk score was significantly different for cases stratified by histological grade (Figure 4B) and KPS (Figure 4C) but not by gender (Figure 4D) and age (Figure 4E). To further investigate the prognostic value of risk score and other clinic pathological features, univariate and multivariate Cox regression analyses were performed both in TCGA and CGGA databases. In TCGA databases, risk score was a prognostic factor independent of age, gender, histological grade, *MGMT* promoter status, and KPS (Table 1). In the CGGA databases, risk score was a prognostic factor independent of age, gender, WHO grade, chemotherapy status, and radiotherapy status (Table 2).

**TABLE 1:** Univariate and multivariate analysis of prognostic parameters in The Cancer Genome Atlas (TCGA) database.

Variable	Univariate analysis		Multivariate analysis	
	HR (95% CI)	*P*-value	HR (95% CI)	*P*-value
Signature score	3.997 (2.195–7.278)	<0.001	3.906 (1.881–8.113)	<0.0001
Age at diagnosis	1.043 (1.064–1.022)	<0.001	1.022 (0.997–1.04)	0.080
Gender	1.052 (0.710–1.559)	0.799		
Subgroup (B vs. C)	0.588 (0.433–1.045)	0.078		
Karnofsky performance score	0.982 (0.965–0.998)	0.032	0.992 (0.973–1.011)	0.396
Histological grade Grade II		0.100		
Grade III	0.249 (0.061–1.018)	0.512		
Grade IV	0.779 (0.495–1.226)	0.280		
MGMT promoter status	0.863 (0.554–1.342)	0.512		

**TABLE 2:** Univariate and multivariate analysis of prognostic parameters in the Chinese Glioma Genome Atlas Network (CGGA) database.

Variable	Univariate analysis		Multivariate analysis	
	HR (95% CI)	*P*-value	HR (95% CI)	*P*-value
Signature score	6.118 (1.999–18.721)	0.002	5.106 (1.508–17.285)	0.009
Age at diagnosis	1.018 (0.993–1.044)	0.155		
Gender	0.769 (0.464–1.275)	0.309		
Histological grade Grade II		0.409		
Grade III	0.362 (0.049–2.657)	0.318		
Grade IV	0.715 (0.354–1.444)	0.350		
Subgroup	0.653 (0.333–1.279)	0.214		
Radiotherapy	0.675 (0.211–2.165)	0.509		
Chemotherapy	3.788 (2.088–6.871)	<0.0001	3.583 (1.978–6.489)	<0.0001

The receiver operating characteristic (ROC) curve analysis showed that risk score had the best efficiency compared with age, histologic grade, and subgroups (Groups B and C) for predicting the 14.5-month and 3-year survival of patients in *IDH*-wild type WHO grade IV gliomas in both TCGA (Figures 4F–G) and CGGA (Supplementary Figures 3C,D) databases. These results indicated that signature risk score has a better accuracy in predicting patient’s prognosis than histological grade and newly update subgroup.

### The Biological Processes Associated With the Risk Signature

**FIGURE 5:**
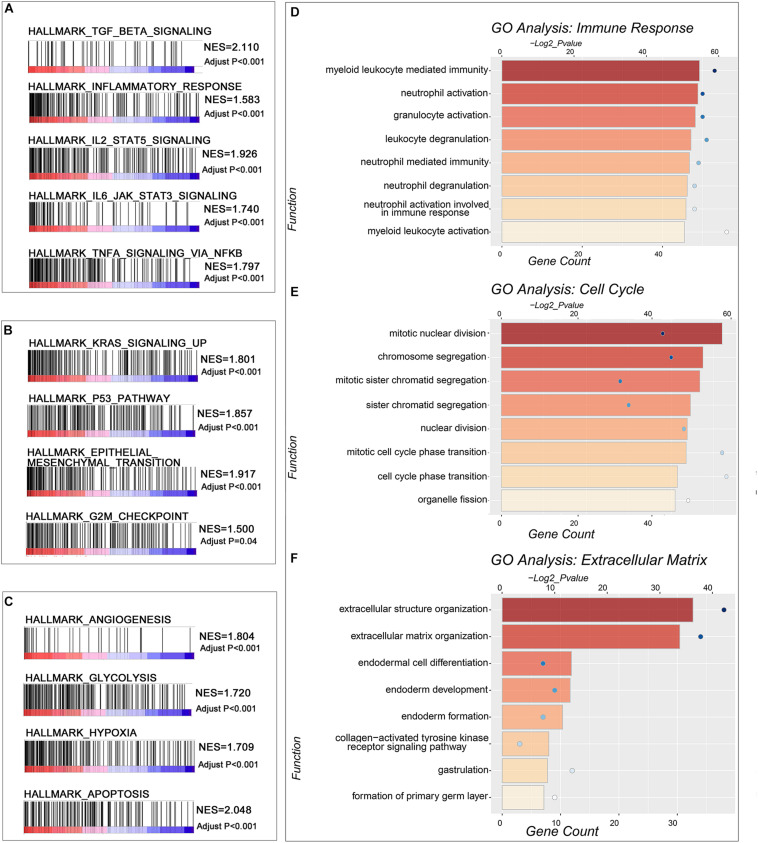
The function annotation of the genes significantly correlated with the risk score. **(A–C)** GSEA revealed hallmarks of malignant tumors positively correlated with *IDH*-wild type WHO grade IV gliomas with high-risk scores. **(D–F)** The GO analysis of the genes positively correlated with the risk score in TCGA database.

It would be helpful for us to understand the clinical significance of the signature and their potential value in guiding therapy to identify the biological functions associated with the signature. Thus, we identified genes positively or negatively correlated with the risk score, and then annotated their functions using GSEA and GO analysis. In the GSEA analysis, the hallmarks related to immune response, transcription regulator, and microenvironment were enriched in the high-risk group (Figures 5A–C). Similar results could be clarified in GO analysis. The results indicated that positively correlated genes were mainly enriched in the biological processes of “immune response,” “cell cycle,” and “extracellular matrix” in both TCGA (Figures 5D–F) and CGGA (Supplementary Figures 4A–C) datasets.

**FIGURE 6:**
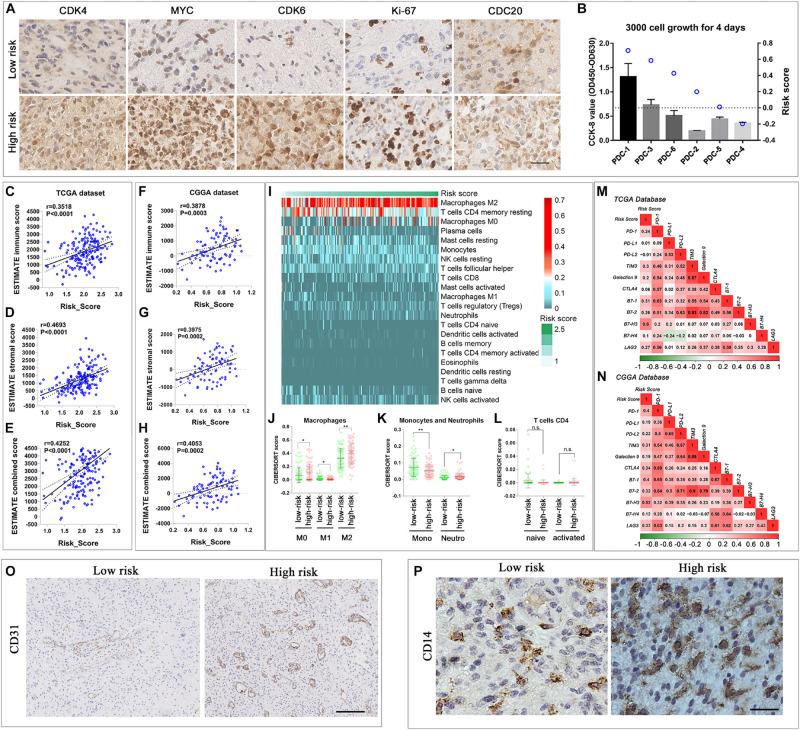
Comparison of malignant biological and immune-related characteristics between cases with low and high risks. **(A)** IHC staining of tissue samples of two different risk score *IDH*-wild type WHO grade IV gliomas. **(B)** CCK-8 proliferation assay of six *IDH*-wild type PDCs and the risk score calculated by RNA-seq data. **(C–H)** Scatterplot showed that immune score, stroma score, and combined score were significantly positively correlated with risk score in TCGA **(C–E)** and CGGA databases **(F–H)**. **(I)** Heatmap described the most enriched infiltrated immune cells in TCGA database arranged by the increasing risk score. **(J–L)** The distribution of the CIBERSORT score of the infiltrated immune cells (macrophage, monocytes, and neutrophils, T-cell CD4) subtypes in low- and high-risk groups in TCGA database. The correlation between the risk score and the expression of classic immune checkpoint genes in TCGA **(M)** and CGGA database **(N)**. **(O)** IHC staining of vessel-related protein, CD31, of two different risk score *IDH*-wild type WHO grade IV gliomas tissue samples (scale bar: 400 um). **(P)** IHC staining of monocytes and macrophage related protein, CD14, of two different risk score *IDH*-wild type WHO grade IV glioma tissue samples (scale bar: 50 um) (**P < 0.01, *P < 0.05, n.s., not significant).

The risk score was correlated with cell cycle. Thus, we used the *IDH*-wild type glioma sample in the CGGA database and PDCs to validate the results of the bioinformatics analyses. In IHC, the G1 phase progression and G1/S transition-related genes *CDK4* and *CDK6*, transcription-related gene *MYC*, and microtubule-dependent process-related gene *CDC20* were elevated in the high-risk score patient sample (Figure 6A). The proportion of Ki-67-positive nuclei was elevated in the high-risk group as well. Meanwhile, the CCK-8 assay was used to evaluate the PDC proliferation. The results showed that high-risk PDCs have a significant higher rate of proliferation than low-risk PDCs (Figure 6B).

Considering the close relationship between the immune-microenvironment and the biological processes of “immune response” and “extracellular matrix,” it is worthwhile to further elucidate the relationship between the signature and immune-microenvironment characteristics of all *IDH*-wild type WHO grade IV gliomas (Groups B and C). First, to analyze the immune and stromal cells of each case, the R package (ESTIMATE) was applied in TCGA (Figures 6C–E) and CGGA database (Figures 6F–H). The results showed that both immune score and stromal score were all positively correlated with the risk score, which indicated that the infiltration of immune cell in glioma increased in the high-risk group.

Thus, we employed the CIBERSORT to estimate the abundance of infiltrated immune cells in gliomas with the different risk score. In TCGA database, “Macrophage M2,” “T-cell CD4 memory resting,” and “Macrophage M0” were ranked as the most infiltrated immune cells (Figure 6I). In the high-risk group, the ratio of “neutrophils,” “Macrophage M0” and “Macrophage M2” increased, while the amount of “monocyte” and “Macrophage M1” were elevated in the low-risk score (Figures 6J,K). Among the CD4-positive T cells, we observed the trend that the naive subtype decreased in the high-risk group, while the memory-activated subtype increased in the high-risk group, although the *P*-value was greater than 0.05 (Figure 6L). In the CGGA database, the amount of “Macrophage M0” and “T-cell CD4 memory activated” was significantly different in the low- and high-risk groups (Supplementary Figures 5A–D). The increase in “Macrophage M0” and “T-cell CD4 memory activated” in the high-risk group reflect the common immune environment variation in both TCGA and CGGA databases.

Immune exhaustion is also a phenomenon in immune environment variation. Thus, we fully tested the immune checkpoint expression. Most immune checkpoint expressions are positively correlated with risk score in TCGA and CGGA databases, which indicated the increasing rate of immune exhaustion (Figures 6M,N). These results indicated that the novel gene signature reflected the *IDH*-wild type WHO grade IV glioma cellular functional characteristics, and the immune microenvironment variation related to the poor prognosis, thus, can predict the survival of *IDH*-wild type WHO grade IV gliomas.

We also verified the bioinformatic analysis results through IHC; the results showed that the expression of vessel protein CD31 was increased in the case with the high-risk score (Figure 6O). Similarly, the expression of CD14, a protein preferentially expressed on monocytes and macrophages, was also increased in the case with the high risk score (Figure 6P).

## Discussion

The integrated diagnosis of Louis et al. (2016) classification and cIMPACT-NOW updates have greatly improved our understanding of the heterogeneity of diffuse gliomas based on their molecular features (Louis et al., 2016; Brat et al., 2018). Currently, *IDH*-wild type gliomas, with relatively poorer survival, could be classified into three groups based on their histologic and genetic features (Brat et al., 2018). Here, we successfully identified the differentially enriched transcriptional characteristics among the three groups of *IDH*-wild type gliomas. Based on the transcriptional characteristics, we also constructed a nine-gene signature from the identified genes to further stratify the *IDH*-wild type WHO grade IV gliomas (including Group B and Group C), whose prognosis cannot be properly stratified by current classification criterion. Finally, the constructed signature could properly stratify prognosis, cell proliferation activities, extracellular matrix-mediated biological activities, and immune-microenvironment of *IDH*-wild type WHO grade IV gliomas in both TCGA and CGGA datasets.

In this study, we figured out that the “cell cycle,” “cell mitosis,” and “DNA replication and repairer”-related biological processes were the major differential biological features between diffuse astrocytic gliomas with different molecular features, and these biological processes were elevated in diffuse astrocytic gliomas with GBM molecular features. An unlimited cell cycle increases DNA damage and genetic instability, which leads to more malignant biomarker changes (Tubbs and Nussenzweig, 2017). *TERT* promoter mutation can significantly promote *TERT* expression, and *TERT* can reactivate the telomerase function, which is required as an impetus in gliomas originated from low-rate self-renewal tissue (Killela et al., 2013). In glioma, the *TERT* promotor mutant is related with the recruitment of the multimeric *GABP* transcription factor, which might regulate a mass of proto-oncogene expression. The physiological function of *EGFR* is to modify epithelial tissue development and angiogenesis (Sigismund et al., 2018). *EGFR* amplification is highly related to TMZ resistance in glioma (Ma et al., 2019; Meng et al., 2020). Combined 7 + /10− has been described as a prognostic hallmark and has a strong correlation with the NF-kB complex and Akt pathway (Baysan et al., 2017). Thus, our finding indicated that biological characteristics identified by transcriptional profile analysis could also reflect genetic alterations in gliomas to some extent.

Compared with diffuse astrocytic gliomas with GBM molecular features, we also observed that microenvironment-related hallmarks, “angiogenesis” and “hypoxia,” and biological processes of “extracellular matrix” and “immune response” were significantly enriched in GBM. The extracellular matrix is a dynamic structure to support tissue integrity and elasticity, responsible for cell homeostasis and angiogenesis (Bonnans et al., 2014; Zhao et al., 2018). The ECM provides essential signals to regulate cell diverse functions to promote malignant tumor invasion (Bissell and Hines, 2011; Bonnans et al., 2014; Ferrer et al., 2018; Chai et al., 2019d). This finding is also consistent with the histologic features of GBM with microvascular proliferation and/or necrosis (Louis et al., 2016). All of these again supported that the transcriptional profile could not only reflect the genetic and histologic features of gliomas but also has the potential to reveal biological heterogeneity of gliomas with similar genetic and histologic features. This is also the basis that we can successfully develop a transcriptional signature to stratify the prognosis and biological features of *IDH*-wild type WHO grade IV gliomas.

The CNS is an immune privilege in traditional thoughts. Nowadays, growing evidence has proved that the prognosis of glioma is closely related to immune response (Topalian et al., 2015; Meng et al., 2019; Aslan et al., 2020). Developing immunotherapy for gliomas has also attracted extensive investigation (Ogbomo et al., 2011; Topalian et al., 2015). Transcriptional characteristics can also reflect the status of tumor immune-microenvironment, which is critical for developing immunotherapy for *IDH*-wild type gliomas (Zhang et al., 2017; Cai et al., 2018; Martinez-Lage et al., 2019; Mohme et al., 2020; Zha et al., 2020). Here, we observed a positive correlation between signature risk scores and the number of immune-infiltrating cells in *IDH*-wild type WHO grade IV gliomas. We found that the majority of immune-infiltrating cells were macrophages and monocytes, which is consistent with previous observations (Martinez-Lage et al., 2019). We also identified that the number of infiltrating neutrophils, M0 and M2 macrophages, increased in gliomas with high-risk scores. Neutrophil infiltration that has been reported could promote GBM cell migration (Liang et al., 2014). M0 and M2 macrophages have been thought to be contributors of the immune-suppression status of gliomas (Komohara et al., 2008; Valeta-Magara et al., 2019). This suggested that the constructed signature may also pick up cases with immune-suppression status. Besides, the signature can also reflect the immune exhaustion status of *IDH*-wild type WHO grade IV gliomas. This is supported by the finding that (1) there was an elevated proportion of T-cell CD4 memory activated in gliomas with high-risk score; (2) most of the immune checkpoint expressions were positively correlated with the risk score.

Overall, our findings highlight that the transcriptional characteristics differed among various groups of *IDH*-wild type gliomas. We also demonstrated that these differentially biological characteristics could not only reflect the histologic and genetic molecular features of *IDH*-wild type gliomas but also have the potential to further stratify the biological heterogeneity of a specific group of *IDH*-wild type gliomas. Finally, we successfully developed a transcriptional signature, which could stratify prognosis, biological activities, and immune-microenvironment status of *IDH*-wild type WHO grade IV gliomas. Although further prospective research is still needed to apply our findings to clinical practice, our findings provide an important advance in our understanding of the transcriptional characteristics of various *IDH*-wild type glioma groups and their potential utility as a stratified tool for *IDH*-wild type WHO grade IV gliomas.

## Data Availability Statement

All datasets generated for this study are included in the article/Supplementary Material.

## Ethics Statement

The studies involving human participants were reviewed and approved by institutional review board of Beijing Tiantan Hospital. Written informed consent for participation was not required for this study in accordance with the national legislation and the institutional requirements. Written informed consent was not obtained from the individual(s) for the publication of any potentially identifiable images or data included in this article.

## Author Contributions

R-CC and Y-ZC conceived and designed the study and performed the analyses and wrote the manuscript. GL, BP, K-NZ, and XZ contributed to the data analysis and the critical reading of the manuscript. R-CC performed IHC staining. Y-ZC performed CCK-8 proliferation assay. R-CC, Z-lJ, and Y-ZW supervised the analyses and contributed to the critical reading of the manuscript. All authors contributed to the article and approved the submitted version.

## Conflict of Interest

The authors declare that the research was conducted in the absence of any commercial or financial relationships that could be construed as a potential conflict of interest.

## Footnotes

1 http://cancergenome.nih.gov/

2 https://metascape.org/gp/index.html

3 https://cibersort.stanford.edu/

4 https://www.r-project.org/

## Supplementary Material

Supplementary Figure 1 **(A–F)** The GO analysis of the DEGs upregulated in Group C compared with Group B in CGGA database.

Supplementary Figure 2 **(A,B)** The GO analysis of the DEGs downregulated in Group B compared with Group A in TCGA database. **(C,D)** The GO analysis of the DEGs downregulated in Group C compared with Group B in TCGA database. **(E,F)** The GO analysis of the DEGs downregulated in Group C compared with Group B in CGGA database.

Supplementary Figure 3 **(A)** LASSO Cox regression model. **(B)** Kaplan–Meier survival analysis of the risk signature in *IDH*-wild type glioma, WHO IV, with the molecular feature of GBM (Group B) in CGGA database. **(C,D)** ROC curves showed the predictive efficiency of risk score, age, histological grade according to Louis et al. (2016) guide and subgroup according to cIMPACT-NOW update three recommendation on 14.5-month and 3-year survival in the CGGA database.

Supplementary Figure 4 The GO analysis of the genes positively correlated with the risk score in CGGA database.

Supplementary Figure 5 **(A)** Heat map described the most enriched infiltrated immune cells in CGGA database arranged by the increasing risk score. **(B,C)** The distribution of the CIBERSORT score of the infiltrated immune cells (macrophage, monocytes, and neutrophils, T cell CD4) in low- and high-risk groups in CGGA database (^∗^*P* < 0.05, n.s., not significant).

Supplementary Table 1 Distribution of clinicopathological features in TCGA and CGGA databases.

Supplementary Table 2 TCGA Group B vs. Group A DEGs.

Supplementary Table 3 TCGA Group C vs. Group B DEGs.

Supplementary Table 4 CGGA Group A vs. Group B DEGs.

Supplementary Table 5 CGGA Group B vs. Group C DEGs.

Supplementary Table 6 characteristics of genes upregulated in GBM (Group B vs. A).

Supplementary Table 7 characteristics of genes upregulated in GBM (Group B vs. C).

Supplementary Table 8 The coefficients of Signature Genes.

Supplementary Table 9 Distribution of clinicopathological features between groups with low- and high-risk in TCGA database.

Supplementary Table 10 Distribution of clinicopathological features between groups with low- and high-risk in CGGA database.

## Abbreviations

CGGA: Chinese Glioma Genome Atlas
cIMPACT: The Consortium to Inform Molecular and Practical Approaches to CNS Tumor Taxonomy
CNS: central nervous system
DEGs: differentially expression genes
EGFR: epidermal growth factor receptor
GSEA: gene set enrichment analysis
GO: Gene Ontology
*IDH*: isocitric dehydrogenase
IHC: immunohistochemistry
KEGG: Kyoto Encyclopedia of Genes and Genomes
KPS: Karnofsky performance score
LASSO: least absolute shrinkage and selection operator
TERT: telomerase reverse transcriptase
TCGA: The Cancer Genome Atlas
WHO: World Health Organization.
