# Supplementary material for: Transcriptional Characteristics of IDH-Wild Type Glioma Subgroups Highlight the Biological Processes Underlying Heterogeneity of IDH-Wild Type WHO Grade IV Gliomas
Source: Front Cell Dev Biol. 2020 Oct 22;8:580464. doi: 10.3389/fcell.2020.580464 (PMC7642517; doi:10.3389/fcell.2020.580464)
Supplement: Supplementary Figure 1 — (A–F) The GO analysis of the DEGs upregulated in Group C compared with Group B in CGGA database. [file Table_1.DOCX]

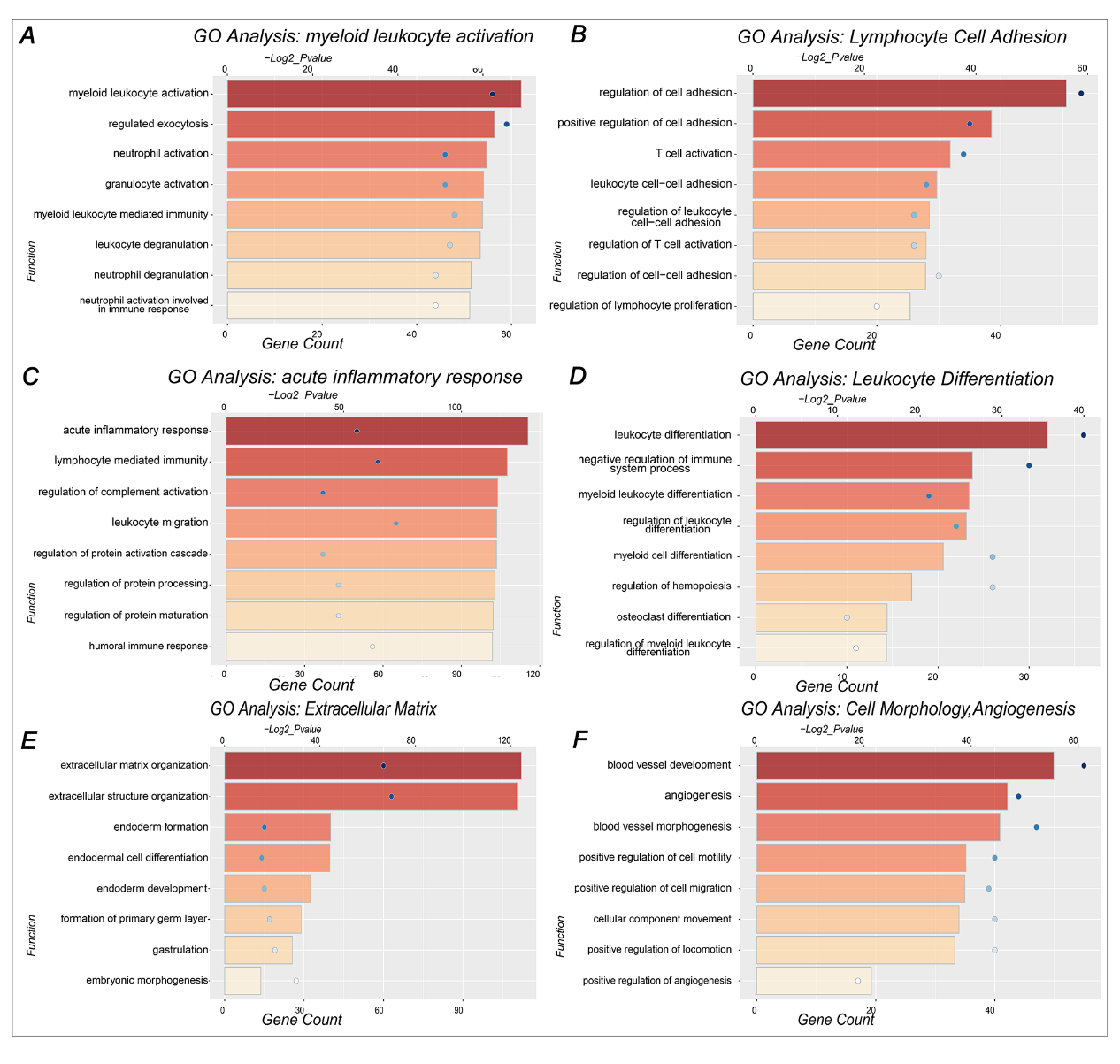


Supplementary Figure 1. (A-F) The GO analysis of the DEGs upregulated in Group C compare with Group B in CGGA database.


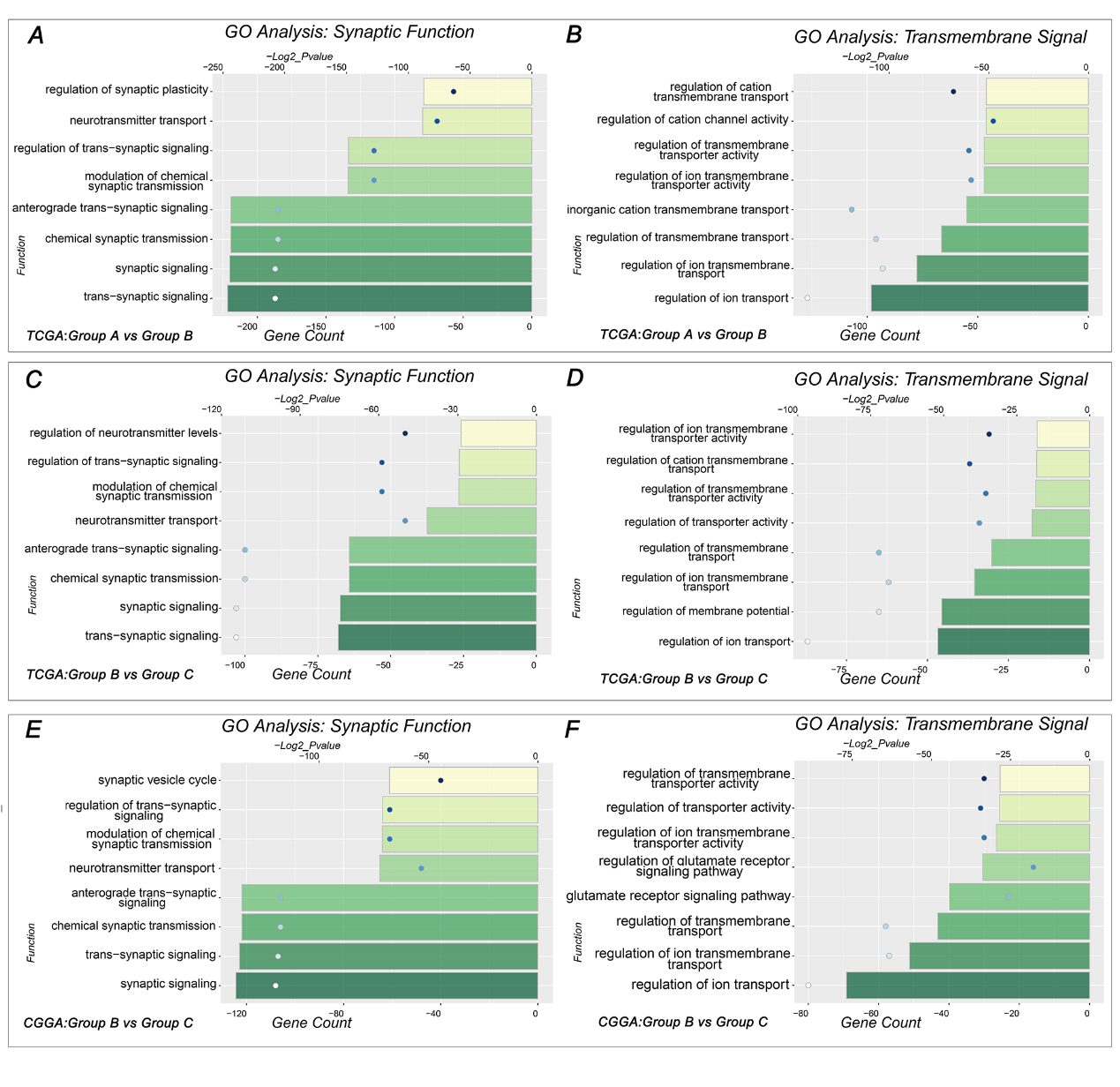


Supplementary Figure 2. (A-B) The GO analysis of the DEGs downregulated in Group B compare with Group A in TCGA database. (C-D) The GO analysis of the DEGs downregulated in Group C compare with Group B in TCGA database. (E-F) The GO analysis of the DEGs downregulated in Group C compare with Group B in CGGA database.


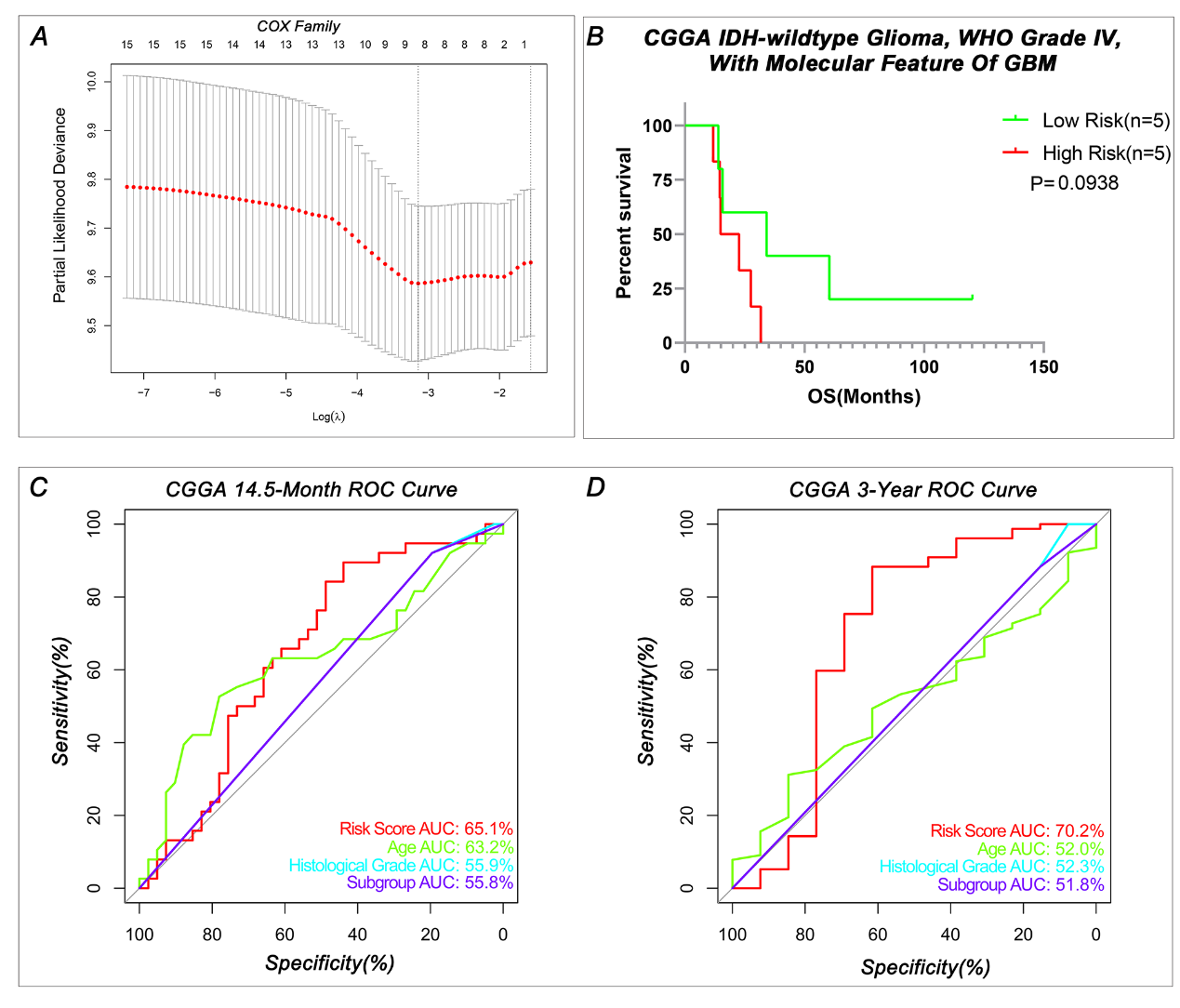


Supplementary Figure 3. (A) LASSO Cox regression model. (B) Kaplan-Meier survival analysis of the risk signature in *IDH*-wildtype glioma, WHO IV, with molecular feature of GBM (Group B) in CGGA database. (C-D) ROC curves showed the predictive efficiency of risk score, age, histological grade according to WHO 2016 guide and subgroup according to cIMPACT-NOW update 3 recommendation on 14.5-month and 3-year survival in the CGGA database.


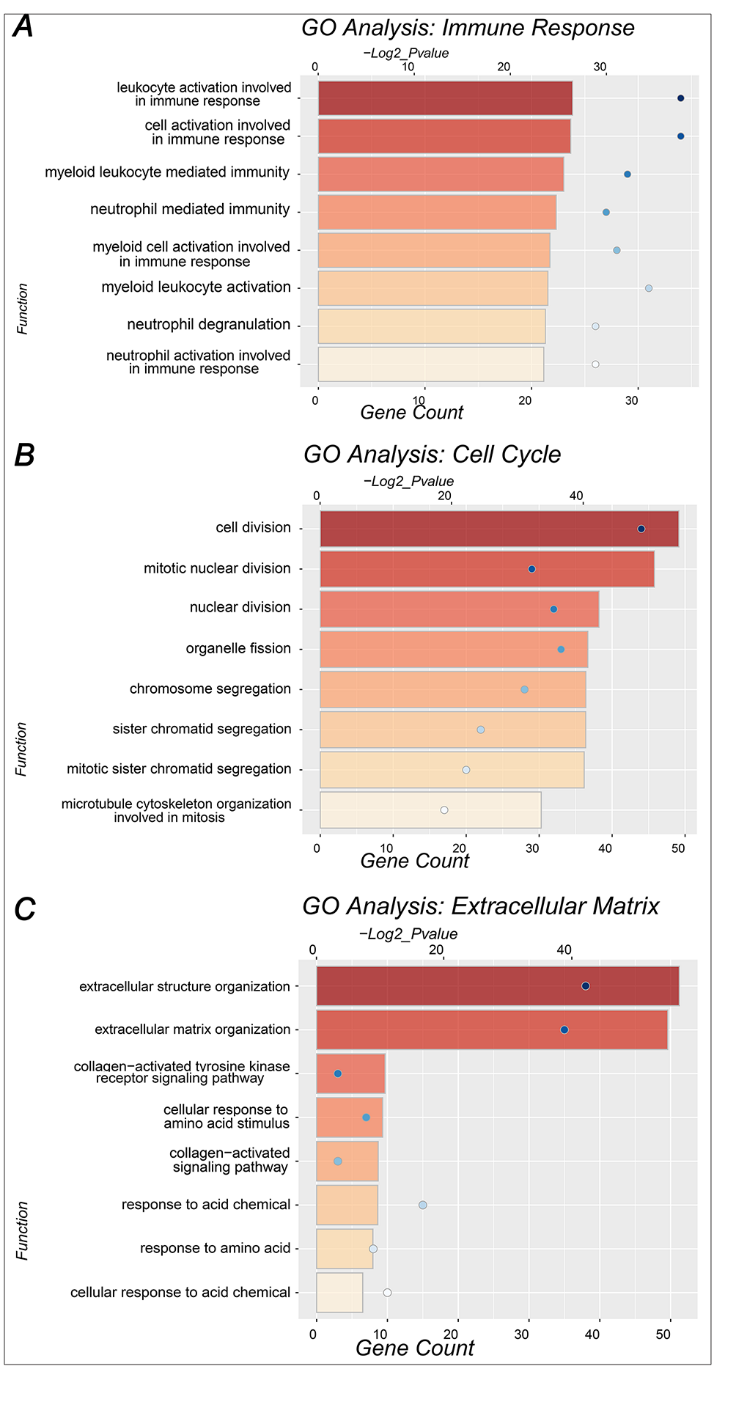


Supplementary Figure 4. The GO analysis of the genes positively correlated with the risk score in CGGA database.


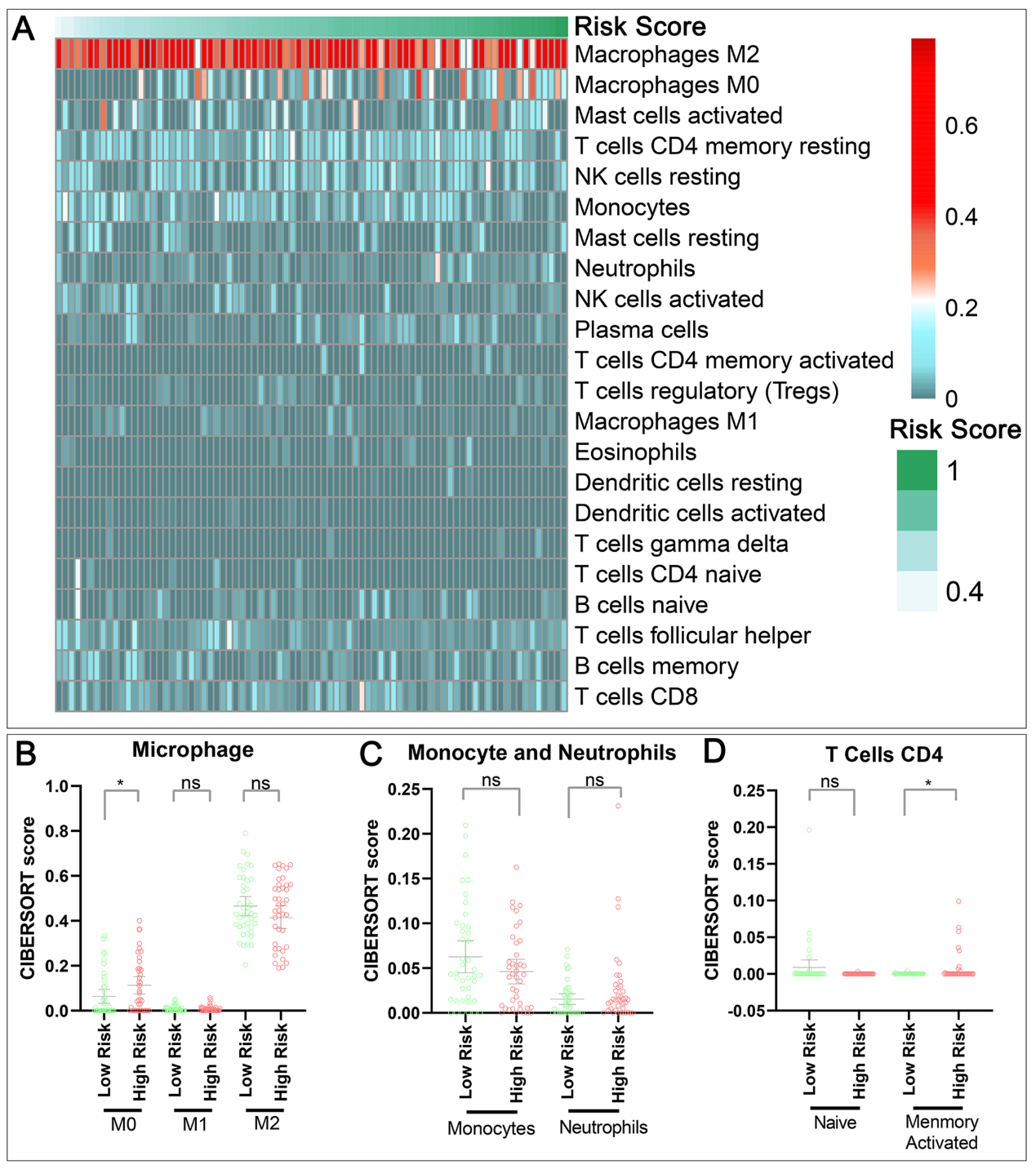


Supplementary Figure 5. (A) Heat map described the most enriched infiltrated immune cells in CGGA database arranged by the increasing risk score. (B-C) The distribution of the CIBERSORT score of the infiltrated immune cells (Macrophage, Monocytes and Neutrophils, T cell CD4) in low- and high-risk group in CGGA database. (* P < 0.05, n.s. not significant)
