# Supplementary material for: Transcriptional Characteristics of IDH-Wild Type Glioma Subgroups Highlight the Biological Processes Underlying Heterogeneity of IDH-Wild Type WHO Grade IV Gliomas
Source: Front Cell Dev Biol. 2020 Oct 22;8:580464. doi: 10.3389/fcell.2020.580464 (PMC7642517; doi:10.3389/fcell.2020.580464)
Supplement: Supplementary Table 1 — Distribution of clinicopathological features in TCGA and CGGA databases. [file Data_Sheet_1.ZIP › Supplementary Table 9 Distribution of clinicopathological features between groups with low- and high-risk in TCGA database.docx]

**Table 2 Distribution of clinicopathological features between groups with low- and high-risk in TCGA database.**

|  | **Low Risk** | | **High Risk** | | **P-value** |
| --- | --- | --- | --- | --- | --- |
| **Grade** |  |  |  |  | <0.0001 |
| II | 6 | 7.0% | 0 | 0.0% |  |
| III  IV | 40  39 | 47.0%  45.8% | 7  78 | 7.0%  91.7% | 0.0327 |
| **KPS score**  ≤80  >80  Unknow | 44  20  21 | 51.8%  23.5%  24.7% | 51  9  25 | 63.5%  10.6%  29.4% |  |
| **Age** |  | 23-87 (59) |  | 36-85 (61) | 0.3061 |
| **Gender** |  |  |  |  | 0.5290 |
| Male | 50 | 58.8% | 54 | 63.5% |  |
| Female | 35 | 41.2% | 31 | 36.5% |  |
| **MGMT** |  |  |  |  | 0.6256 |
| Methylation | 35 | 41.2% | 27 | 31.8% |  |
| Unmethylation  Unknow | 44  6 | 51.8%  7% | 40  18 | 47.0%  21.2% |  |
| **TERT promoter** |  |  |  |  | 0.2302 |
| Mutant | 49 | 57.6% | 18 | 21.2% |  |
| Wildtype | 4 | 4.7% | 0 | 0% |  |
| Unknow | 42 | 49.4% | 67 | 78.8% |  |
| **EGFR Status** |  |  |  |  | 0.8763 |
| No Alteration | 39 | 45.9% | 38 | 44.7% |  |
| Amplification | 44 | 51.8% | 45 | 52.9% |  |
| Unknow | 2 | 2.3% | 2 | 2.3% |  |
| **Chr 7 gain/Chr 10 loss** |  |  |  |  | 0.5951 |
| Gain chr 7 & loss chr 10 | 63 | 74.1% | 60 | 70.6% |  |
| No combined CNA | 20 | 23.5% | 23 | 27.0% |  |
| Unknow | 2 | 2.3% | 2 | 2.3% |  |
|  |  |  |  |  |  |
|  |  |  |  |  |  |
