# Supplementary material for: Transcriptional Characteristics of IDH-Wild Type Glioma Subgroups Highlight the Biological Processes Underlying Heterogeneity of IDH-Wild Type WHO Grade IV Gliomas
Source: Front Cell Dev Biol. 2020 Oct 22;8:580464. doi: 10.3389/fcell.2020.580464 (PMC7642517; doi:10.3389/fcell.2020.580464)
Supplement: Supplementary Table 1 — Distribution of clinicopathological features in TCGA and CGGA databases. [file Data_Sheet_1.ZIP › Supplementary Table 10 Distribution of clinicopathological features between groups with low- and high-risk in CGGA database.docx]

**Table 3. Distribution of clinicopathological features between groups with low- and high-risk in CGGA database.**

|  | **Low Risk** | | **High Risk** | | **P-value** |
| --- | --- | --- | --- | --- | --- |
| **Grade** |  |  |  |  | 0.0157 |
| II | 1 | 2.4% | 0 | 0.0% |  |
| III  IV | 9  31 | 21.9%  75.6% | 1  39 | 2.5%  97.5% |  |
| **Age** | 23-74 (53) |  | 32-79 (55) |  | 0.2302 |
| **Gender** |  |  |  |  | 0.4364 |
| Male | 28 | 68.3% | 24 | 60.0% |  |
| Female | 13 | 31.7% | 16 | 40.0% |  |
| **TERT promoter** |  |  |  |  |  |
| Mutant | 10 | 24.3% | 1 | 2.5% |  |
| Wildtype | 0 | 0% | 0 | 0% |  |
| Unknow | 31 | 75.7% | 39 | 97.5% |  |
| **Chemotherapy Status** |  |  |  |  | 0.0366 |
| Received | 32 | 78.0% | 23 | 57.5% |  |
| Non- Received | 7 | 17.1% | 15 | 37.5% |  |
| Unknow | 2 | 4.9% | 2 | 5% |  |
| **Radiotherapy Status** |  |  |  |  | 0.6223 |
| Received | 37 | 90.2% | 35 | 87.5% |  |
| Non- Received | 2 | 4.8% | 3 | 7.5% |  |
| Unknow | 2 | 4.8% | 2 | 5% |  |
|  |  |  |  |  |  |
|  |  |  |  |  |  |
