# Supplementary material for: Transcriptional Characteristics of IDH-Wild Type Glioma Subgroups Highlight the Biological Processes Underlying Heterogeneity of IDH-Wild Type WHO Grade IV Gliomas
Source: Front Cell Dev Biol. 2020 Oct 22;8:580464. doi: 10.3389/fcell.2020.580464 (PMC7642517; doi:10.3389/fcell.2020.580464)
Supplement: Supplementary Table 1 — Distribution of clinicopathological features in TCGA and CGGA databases. [file Data_Sheet_1.ZIP › Supplementary Table 1 Distribution of clinicopathological features in TCGA and CGGA databases.docx]

**Supplementary Table 1 Distribution of clinicopathological features in TCGA and CGGA databases**

|  | **TCGA database** | | **CGGA database** | |
| --- | --- | --- | --- | --- |
| **Total**  **Grade** | 224(100%) | | 99(100%) | |
| II | 19 | 8.5% | 4 | 6.8% |
| III  IV | 66  139 | 29.4%  62.1% | 23  72 | 23.3%  69.9% |
|  |  |  |  |  |
| **Age**  <median  ≥median | 21-89 (59)  99 44.1%  125 55.8% | | 20-79(51)  46 48.8%  53 51.2% | |
|  |  | |  | |
| **Gender** |  |  |  |  |
| Male | 135 | 60.3% | 66 | 62.4% |
| Female | 89 | 39.7% | 33 | 37.6% |
|  |  |  |  |  |
|  |  |  |  |  |
